# Redefining structural stability in Ni-rich single-crystalline cathodes

**DOI:** 10.1093/nsr/nwag292

**Published:** 2026-05-18

**Authors:** Yufeng Yan, Jiaxin Ma, Sang-Young Lee, Zhong-Shuai Wu

**Affiliations:** State Key Laboratory of Catalysis, Dalian Institute of Chemical Physics, Chinese Academy of Sciences, China; Center of Materials Science and Optoelectronics Engineering, University of Chinese Academy of Sciences, China; State Key Laboratory of Catalysis, Dalian Institute of Chemical Physics, Chinese Academy of Sciences, China; School of Materials Science and Engineering, Zhengzhou University, China; Department of Chemical and Biomolecular Engineering, Yonsei University, Republic of Korea; State Key Laboratory of Catalysis, Dalian Institute of Chemical Physics, Chinese Academy of Sciences, China; Center of Materials Science and Optoelectronics Engineering, University of Chinese Academy of Sciences, China

Nickel-rich (Ni-rich) layered cathode materials underpin the drive toward higher-energy-density lithium-ion batteries, yet their practical deployment remains fundamentally limited by structural instability under deep delithiation [1]. Single-crystalline Ni-rich cathodes mark a pivotal advance because eliminating grain boundaries suppresses intergranular cracking and mechanical pulverization [2]. However, mounting evidence indicates that single crystallinity alone cannot resolve the intrinsic instability that emerges under high-voltage cycling: failure increasingly originates within the lattice, where anisotropic phase transitions trigger strain accumulation and progressive structural fatigue. Accordingly, the long-term stability of single-crystalline cathodes is dictated less by particle-level integrity than by the strain accommodation and dissipation at the lattice scale [3].

**Figure 1.**
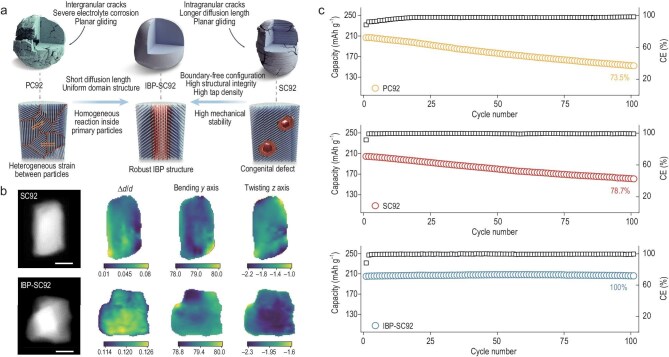
Lattice-strain regulation and electrochemical superiority of intralattice-bonded phase engineered ultrahigh-Ni single-crystalline cathodes. (a) Schematic illustration of the preparation process of the ultrahigh-Ni layered cathodes assisted by the intralattice-bonded phase. (b) XRF images and lattice distortion nanodiffraction mapping of SC92 and IBP-SC92. (c) Cycling stability of PC92, SC92 and IBP-SC92 in coin-type half cells within a voltage window of 2.7–4.3 V at a current rate of 0.5 C with a mass loading of 2–3 mg cm^−2^. Reproduced with permission from Amine *et al.* [4].

In this context, recent work by Amine *et al*. reframes the stability problem for Ni-rich single-crystalline cathodes (SC92) [4] by introducing a lattice-centric design strategy, intrinsic lattice-strain regulation, rather than conventional morphology engineering or surface modification. Through boron (B) and niobium (Nb) co-doping enabled by elevated sintering temperatures and prolonged calcination, an intralattice-bonded phase (IBP) is formed, which interpenetrates the Ni-rich framework to generate a commensurate superstructure (IBP-SC92, Fig. 1a). Functionally, the IBP serves as an internal structural backbone that resists anisotropic contraction during high-voltage operation. Importantly, the study pinpoints the dominant origin of instability in Ni-rich single-crystalline cathodes as the abrupt lattice response during high-voltage phase transitions, manifested by severe interlayer contraction and transition metal–oxygen bond distortion, rather than surface reactivity alone.

Multiscale characterization further shows that IBP engineering reprograms the lattice response during electrochemical cycling. X-ray fluorescence (XRF) and synchrotron X-ray nanodiffraction mapping resolve spatial lattice strain distributions within individual particles, revealing that pristine SC92 accumulates severe and heterogeneous lattice strain, whereas IBP-SC92 maintains a markedly more homogeneous strain field, consistent with efficient stress dissipation by the internal backbone (Fig. 1b). At the atomic scale, the reinforced transition metal–oxygen framework constrains excessive bond distortion and suppresses interslab gliding, enabling IBP-SC92 to retain mechanical integrity without intragranular cracking after prolonged cycling. Complementarily, *in-situ* differential electrochemical mass spectrometry (DEMS) shows substantially suppressed O_2_ and CO_2_ evolution for IBP-SC92 relative to pristine SC92, underscoring enhanced structural resilience under deep delithiation [5].

This lattice-scale stabilization translates directly into electrochemical durability: under aggressive cycling, the IBP-engineered cathode exhibits near-zero capacity fading, delivering 100% retention over 100 cycles in half cells while mitigating voltage decay, markedly outperforming both pristine Ni-rich polycrystalline (PC92) and single-crystalline (SC92) cathodes (Fig. 1c). Notably, the improvement is achieved without sacrificing deep delithiation, indicating that the gain arises from intrinsic lattice stabilization rather than kinetic or surface-limited effects.

In summary, this study demonstrates that intralattice-bonded phase engineering provides a mechanistic route to stabilize ultrahigh-Ni single-crystalline cathodes via direct modulation of lattice-scale strain evolution. Such a strategy complements conventional morphology-based approaches, revealing that structural integrity can be enhanced not only at the particle level but also at the lattice scale. Consequently, IBP engineering emerges as a rational and adaptable design principle for stabilizing next-generation high-energy cathode materials and enabling durable, high-performance lithium-ion batteries.
